# The Taxonomy Statistic Uncovers Novel Clinical Patterns in a Population of Ischemic Stroke Patients

**DOI:** 10.1371/journal.pone.0069816

**Published:** 2013-07-16

**Authors:** Andrzej Tukiendorf, Radosław Kaźmierski, Sławomir Michalak

**Affiliations:** 1 Department of Epidemiology, Cancer Center-Institute of Oncology, Gliwice, Poland; 2 Department of Neurology and Cerebrovascular Disorders, Poznań University of Medical Sciences, Poznań, Poland; 3 Department of Neurochemistry and Neuropathology, Poznań University of Medical Sciences, Poznań, Poland; Queen's University Belfast, United Kingdom

## Abstract

In this paper, we describe a simple taxonomic approach for clinical data mining elaborated by Marczewski and Steinhaus (M-S), whose performance equals the advanced statistical methodology known as the expectation-maximization (E-M) algorithm. We tested these two methods on a cohort of ischemic stroke patients. The comparison of both methods revealed strong agreement. Direct agreement between M-S and E-M classifications reached 83%, while Cohen’s coefficient of agreement was κ = 0.766(*P* < 0.0001). The statistical analysis conducted and the outcomes obtained in this paper revealed novel clinical patterns in ischemic stroke patients. The aim of the study was to evaluate the clinical usefulness of Marczewski-Steinhaus’ taxonomic approach as a tool for the detection of novel patterns of data in ischemic stroke patients and the prediction of disease outcome. In terms of the identification of fairly frequent types of stroke patients using their age, National Institutes of Health Stroke Scale (NIHSS), and diabetes mellitus (DM) status, when dealing with rough characteristics of patients, four particular types of patients are recognized, which cannot be identified by means of routine clinical methods. Following the obtained taxonomical outcomes, the strong correlation between the health status at moment of admission to emergency department (ED) and the subsequent recovery of patients is established. Moreover, popularization and simplification of the ideas of advanced mathematicians may provide an unconventional explorative platform for clinical problems.

## Introduction

Epidemiological studies focused on disease etiology generally use methods that lead to the identification of risk factors via the calculation of odds ratios, correlations and regression analyses of variables. To develop statistical methodology for use in clinical research and public health, taxonomy can widen existing exploratory tools. This tool seems to offer a unique method for exploring epidemiological findings. In this paper, we demonstrate its effectiveness.

From the Greek ‘*taxis*’ = arrangement/division and ‘*nomos*’ = law/method, taxonomy has, in the past, usually referred to the classification of biological systems. The first taxonomic system is attributed to Aristotle and his book *On the Parts of Animals* [1]. Aristotle is assumed to be the earliest ancient inventor of the concept of ranked classification. One of the best-known new-era biological taxonomies was devised by Carolus Linnaeus, whose classification *Systema Naturæ*, 10th edition [2], has had an enormous impact on science (it is still widely applied in a modified manner). However, neither of these men invented or gave taxonomy its contemporary form.

A modern statistical taxonomical approach was first proposed by Tryon [3]. His analysis, termed cluster analysis, forms groups of objects (clusters) by minimizing within-group variance and maximizing between-group variance [3]. Clusters are organized by supertype-subtype/parent–child relationships, which depend on measures of similarity; the technique relies on linking more and more objects together and aggregating larger and larger clusters of increasingly dissimilar elements [3].

Taxonomy uses a wide range of algorithms to determine the distance between objects. In clinical studies, the objects are represented by single patients, with their characteristics described by a number of variables. The most straightforward way of computing distances between objects in a multi-dimensional space is to compute Euclidean distances using the Pythagorean formula. Using this formula for distance, Euclidean space becomes a metric space (Euclidean distances are computed from raw data and not from standardized data). Among a wide set of taxonomical metrics, a Mahalanobis distance [4] is also widely used in cluster analysis. It is based on correlations between variables, through which different patterns can be identified (it differs from Euclidean distance because it takes into account the correlations of the data set and is scale invariant, i.e., not dependent on the scale of measurements because the variables are normalized).

When the distances between the objects are defined by the chosen measure, a linkage rule is used to determine when two clusters are sufficiently similar to be linked together. There are various possibilities of linkage methods. Among the most common techniques are single and complete linkages. For example, in the single linkage method, the smallest dissimilarity between objects in different clusters is used, while in complete linkage, the largest dissimilarity between objects is taken into account.

Following recent definitions, taxonomy is the theoretical study of the classification of empirical entities [5]. When the objects are structured in clusters, they are ‘ready’ to be interpreted, i.e., classification trees can be used to explain the membership of objects in the clusters and their underlying predicting factors. This method may provide an alternative explorative platform for the identification of such predictors.

In light of this brief theoretical background of taxonomy, an original metric (distance) was proposed by Edward Marczewski *vel* Szpilrajn and Hugo Steinhaus [6], which relies on the use of a symmetric difference between objects. In its simplest idea, the taxonomic distance (D) of objects (A, B) is defined as follows D = |A–B|/max(A, B), where nominator is the modulus of A–B, and denominator is the maximum of A and B. The idea was also subsequently highlighted by Stanisław Marcin Ulam, who was Steinhaus’ friend and co-operator of the famous *Scottish Café* as well as a *Manhattan Project* member [7]. Some arithmetic examples of the application of the Marczewski-Steinhaus (M-S) metric are given in the last section of their paper [6]. The proponents of the idea were hopefully not mistaken in arguing that “the distance seems to be useful in several practical applications and especially in some biological problems” [6].

We have undertaken the present study to analyze the clinical usefulness of Marczewski-Steinhaus’ taxonomic approach as a tool for the detection of novel patterns of data. To demonstrate a practical application of the method, we used an example dataset of ischemic stroke patients. Additionally, to predict the outcome in this group of patients, we created a user package for the analysis using the taxonomic method.

## Materials and Methods

The study included 602 ischemic stroke patients (this group of patients was described in previously detail [8,9]). Briefly, all subjects were diagnosed at the emergency departments (EDs) and stroke units of regional and university hospitals. The initial evaluation on admission included medical history, head computerized tomography, laboratory examinations and neurological examination, including the quantification of neurologic deficit using the National Institutes of Health Stroke Scale (NIHSS) score [10]; long-term follow-up of patients was carried out with the use of the modified Rankin scale (mRS) [11] and Barthel index (BI) [12] to determine the functional status of each surviving patient. Outcome measurements were assessed with mRS and BI scores on the 30^th^, 90^th^, 180^th^ and 360^th^ day after the stroke [8,9]. In 31% (n=188) of patients, a positive history of diabetes mellitus (DM) was noted or a *de novo* diagnosis made.

The three parameters considered in the taxonomic method included age, NIHSS score and DM presence. The choice of dataset in this method is up to the researcher; however, factors that showed at least some level of significance should be considered. Additionally, our choice was motivated by methods used in other reports [13–16].

Because the data differ in absolute values, it was necessary to normalize them into the 0–1 range. After normalization, a distance matrix was created in an Excel spreadsheet, which was then used to build a classification tree. In our case, the dendrogram (based on complete linkage) was built in the ‘*cluster*’ package [17] of the R software [18] (the statistical procedures are readily available to the Readers as a link from the PLoS ONE website – open the File S1). Finally, a one-way analysis of variance (ANOVA) was conducted to study the group (type) effects.

To verify the taxonomic method, a parallel statistical analysis was conducted, i.e., the so-called expectation-maximization (E-M) algorithm [19]. The E-M algorithm is widely used for clustering, classification, and density estimation results, and the choice of its application in our study was motivated by its high degree of complexity (in comparison to the M-S algorithm), popularity and reliability (it is ranked 11^th^ among the most cited statistical papers in the world [20]). Particularly, it is an iteration method which alternates between the expectation (E) step, which generates a function for the expectation of the log-likelihood evaluated using the current estimate for the parameters, and the maximization (M) step, which estimates parameters maximizing the expected log-likelihood found on the E step. Intuitively, what E-M does is iteratively ‘augment’ the data by ‘guessing’ the values of the hidden variables and re-estimating the parameters by assuming that the guessed values are the true values [21]. For a basic statistical operation, we adopted an R software package called ‘*mclust*’ [22]. Following the earlier results obtained using the M-S algorithm, we assumed the number of components in the Gaussian mixture (or simply the number of possible clusters) to be equal to four (‘*G*’ argument – see 22 for details).

## Results

### Marczewski-Steinhaus Algorithm

**Figure 1:**
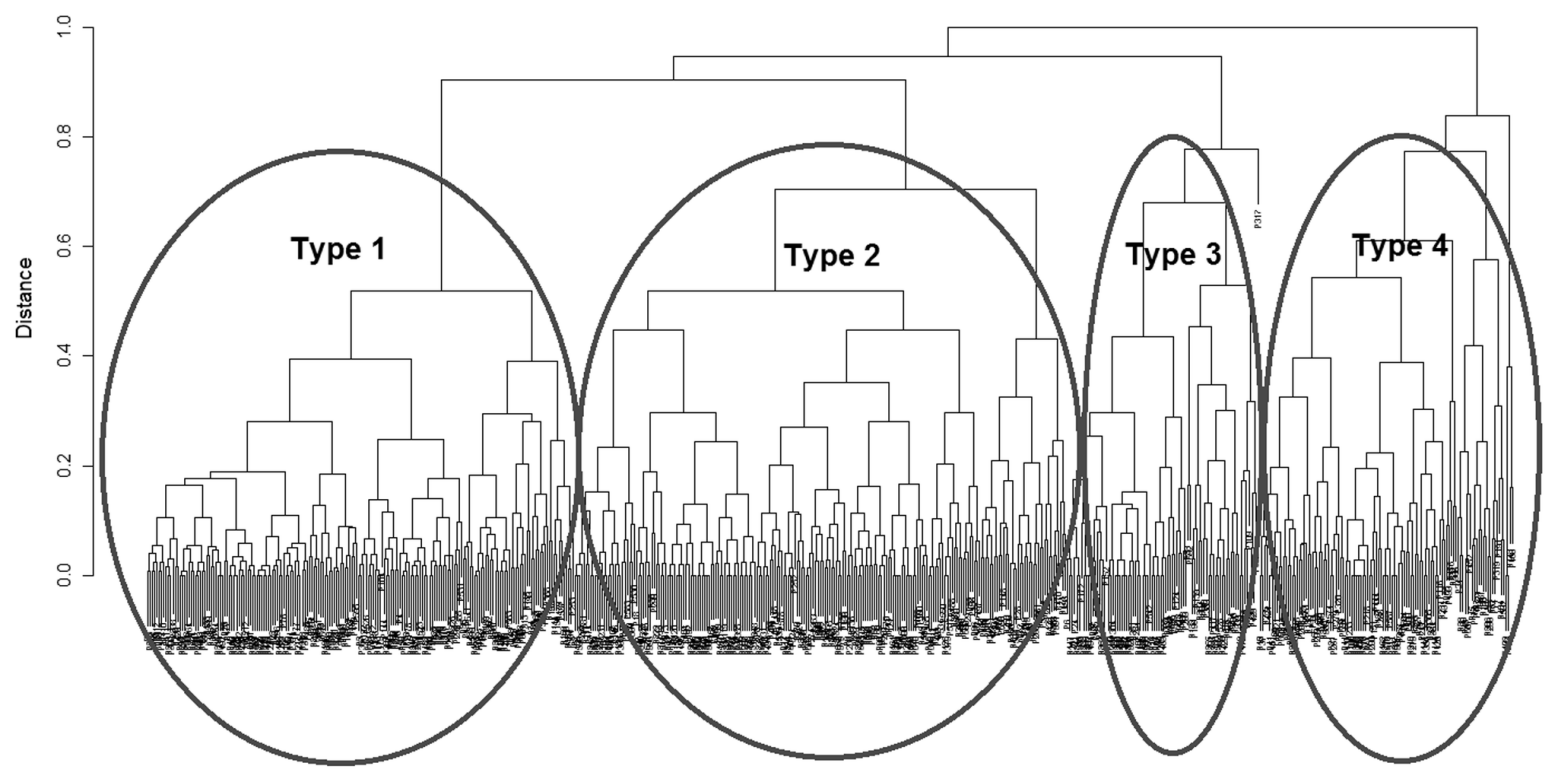
Classification tree of patients.

A resulting classification tree is presented in Figure 1.

The dendrogram shown in Figure 1 provides evidence of four main types of patients (marked in ovals), ordered from the lowest (Type 1) to the highest (Type 4) variability of distances. Next, a re-analysis of the achieved clusters using BI and mRS scores was conducted.

Statistical characteristics of the types of patients based on E-M classification, together with the *F* statistic and *P*s (following one-way ANOVA), are presented in Table 1.

**Table 1:** Characteristics of types of stroke patients (following use of the M-S algorithm).

Types	Age mean (SD)	NIHSS mean (SD)	DM status	# of patients
1	68.7 (10.7)	7.8 (6.6)	positive	188
2	77.9 (7.7)	7.6 (7.0)	negative	217
3	55.5 (5.7)	2.2 (1.6)	negative	85
4	57.0 (8.8)	12.3 (6.4)	negative	112
*F* statistic	212.5	42.3	n/a	
*P*	< 0.0001	< 0.0001	n/a	

**Figure 2:**
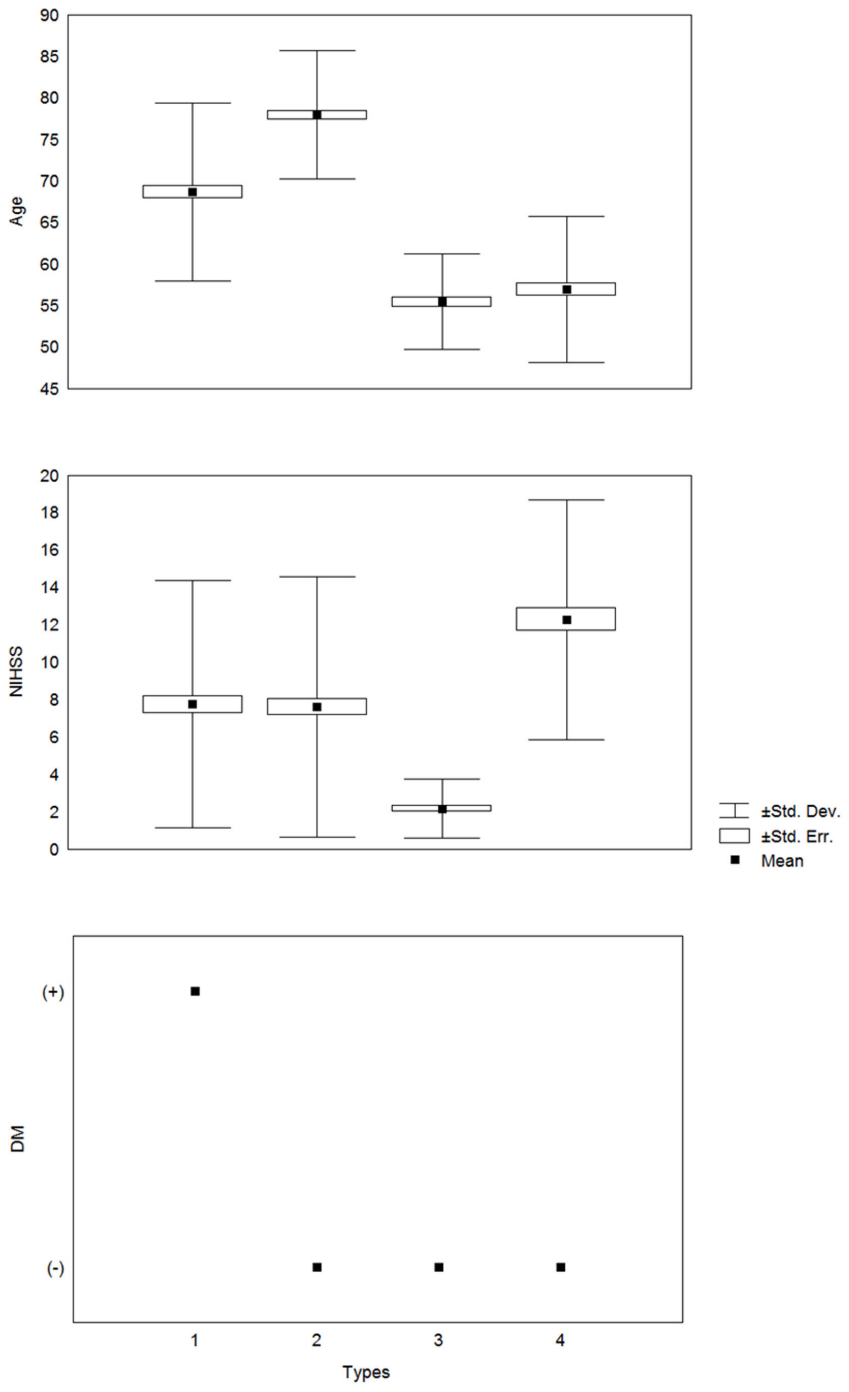
Characteristics of stroke patients following M-S classification (for age, NIHSS score, and DM status).

The results reported in Table 1 show statistically significant differences between the taxonomical types of stroke patients in terms of age of patients and NIHSS; the estimated *F* statistics and *P*s are shown in Table 1. The established differences are presented in a combined plot in Figure 2.

A rough analysis of the characteristics of patients shown in Figure 2 indicates that there are four ‘specific’ types of stroke patients (Table 2).

**Table 2:** Rough approximation of stroke patients (following M-S classification).

Types	Age	NIHSS	DM status
1	medium	medium	positive
2	older	medium	negative
3	younger	lower	negative
4	younger	higher	negative

It is noteworthy that accounting for the total number of combinations for the assumed categories of age of patients (i.e., younger, medium, and older), NIHSS score (i.e., lower, medium, and higher) and DM status (i.e., negative, and positive), we should have 3*3*2 = 18 ‘specific’ types of stroke patients admitted to the stroke units. Therefore, the remaining 14 types of patients were ‘missing’ in our study. Additional results were obtained from Tables 1 and 2 and detailed below. Both Type 1 and 2 patients have nearly identical NIHSS score means and variations (see Table 1 and Figure 2 for details) but differ by DM status; therefore, the difference in age of 78–69 = 9 years (we can approximate to a decade) at this stage of life is equivalent to the DM-positive status in patients.

From a medical point of view, Type 4 patients seem to be somewhat ‘suspicious’: young patients with negative DM status apparently manifest higher NIHSS scores. Most likely, other risk factors, including larger size or a less favorable localization of stroke, underlie these patients’ elevated NIHSS score. However, a more precise analysis of this question is not within the scope of this paper. Nevertheless, a taxonomical approach could be a useful statistical tool for identifying unspecified underlying causes of the health status of patients.

The follow-up outcomes in patients at 30, 90, 180, and 360 days since onset of stroke are detailed in Table 3.

**Table 3:** Means of follow-up outcomes (based on M-S classification).

Scale	Barthel mean (SD)				mRankin mean (SD)			
Types/Days	30	90	180	360	30	90	180	360
1	61.1 (39.8)	61.3 (40.4)	60.6 (41.0)	55.5 (43.6)	3.0 (2.2)	3.0 (2.2)	3.0 (2.3)	3.3 (2.4)
2	63.4 (39.1)	65.6 (40.8)	63.7 (42.2)	58.2 (43.6)	2.8 (2.2)	2.6 (2.4)	2.7 (2.4)	3.0 (2.4)
3	94.0 (12.8)	96.1 (9.5)	96.2 (11.4)	95.2 (13.5)	0.9 (1.4)	0.7 (1.2)	0.6 (1.2)	0.7 (1.3)
4	56.0 (39.0)	62.3 (39.6)	63.8 (40.5)	59.7 (43.1)	3.2 (2.1)	2.9 (2.2)	2.8 (2.4)	3.0 (2.5)
*F* statistic	18.7	17.4	17.1	15.5	22.5	23.6	23.2	20.5
*P*	< 0.0001	< 0.0001	< 0.0001	< 0.0001	< 0.0001	< 0.0001	< 0.0001	< 0.0001

**Figure 3:**
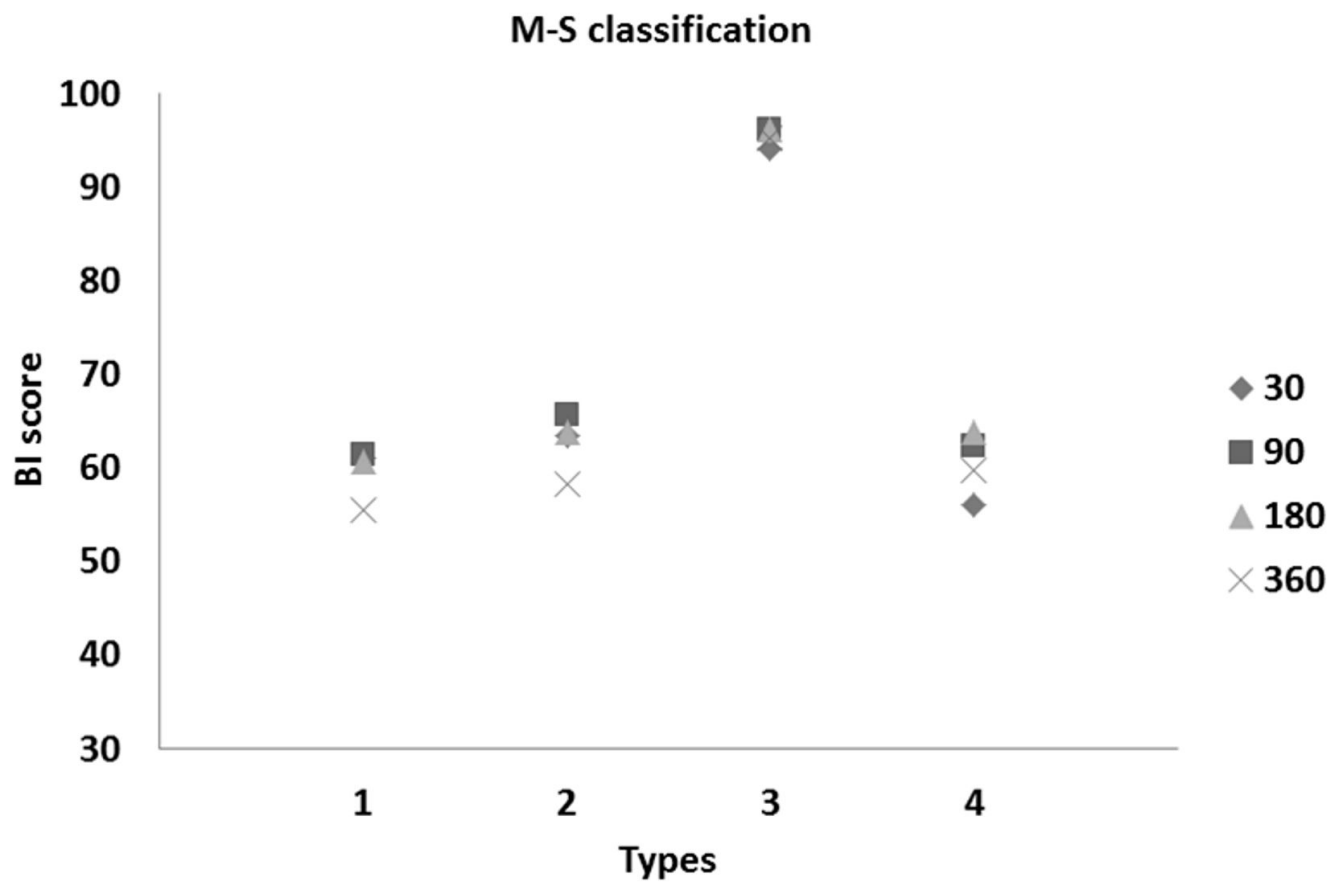
Means of BI scores vs. types of stroke patients at 30, 90, 180, and 360 days after onset of stroke (following M-S classification).

**Figure 4:**
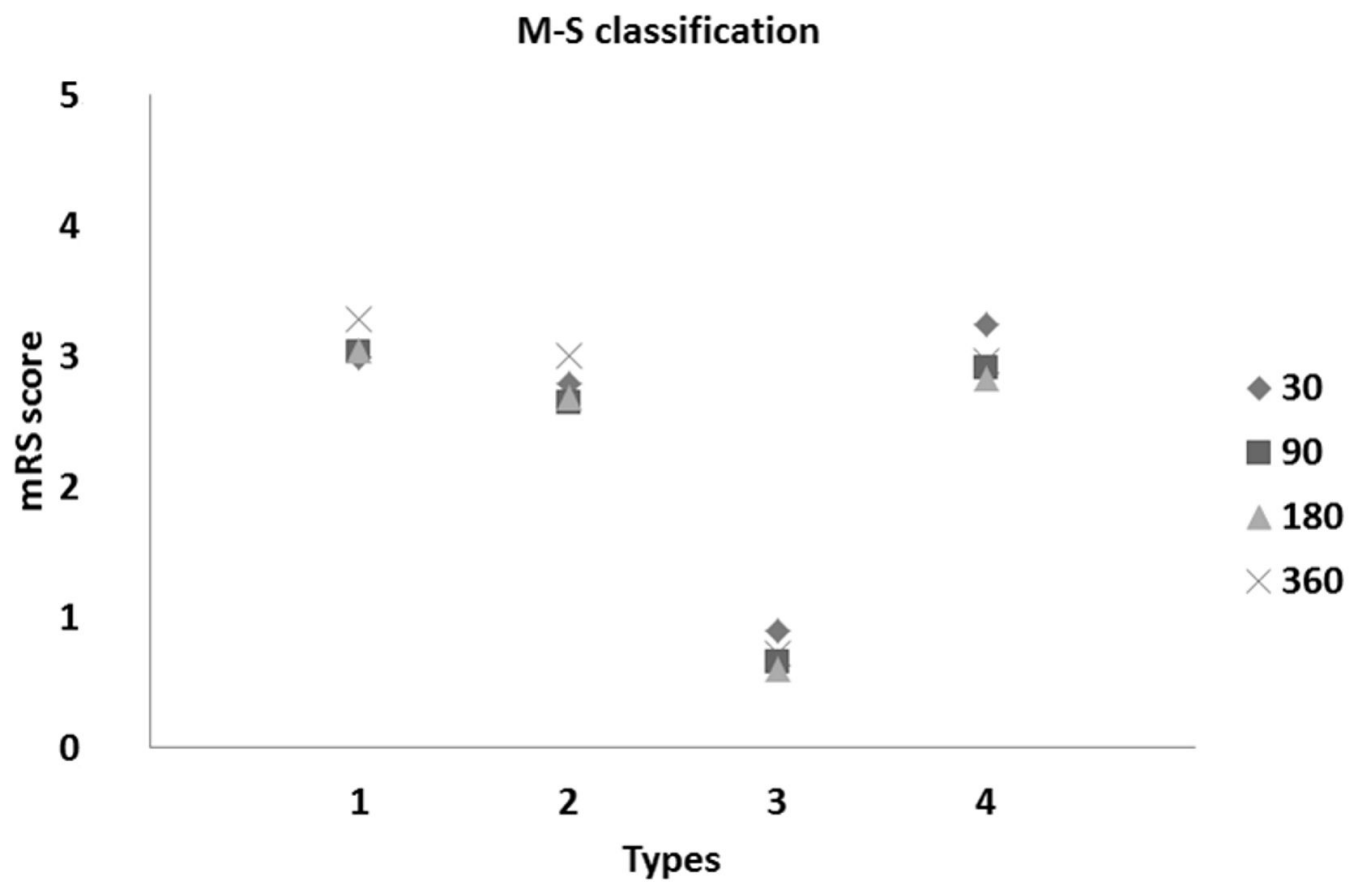
Means of mRankin’s scores vs. Types of stroke patients at 30, 90, 180, and 360 days after onset of stroke (following M-S classification).

We found a significant difference between the taxonomical types of patients during the follow-up of stroke onset up to 360 days (both for the BI and mRS scores). Plots of these patients are depicted in Figures 3 and 4.

Based on the obtained results (Table 3 and Figures 3 and 4), we observed roughly similar trends of disability measures in taxonomical types of stroke patients. Moreover, we noted strong similarity between the means of BI and mRS scores within the established types (see Figures 3 and 4). Roughly evaluated disability levels (or unfavorable outcomes) for the determined types of patients in the follow-up observations are reported in Table 4.

**Table 4:** Disability levels (unfavorable outcomes) in types of stroke patients.

Types	Disability
1	higher
2	higher
3	lower
4	higher

Based on the classification shown in Table 4, we observed that the best health status after onset was predicted for Type 3 patients (i.e., 85/602 = 14.1%). The other types had worse prognoses within the first year of observation. One of the other scientific speculations that can be made from the obtained results is as follows.

Because the follow-up outcomes for Type 2 patients are close to those of Type 4 patients and because both populations are DM negative, the difference in age between these groups (approximately two decades (78–57 = 21 years) is equivalent to nearly five points in NIHSS score (12.3–7.6 = 4.7, see Table 1 for details). As a consequence, the ratio of 20/5 predicts a clinical deterioration of approximately 1 point in the NIHSS score per 4 years of life, starting from ages in the late fifties.

## Expectation-Maximization Algorithm

**Figure 5:**
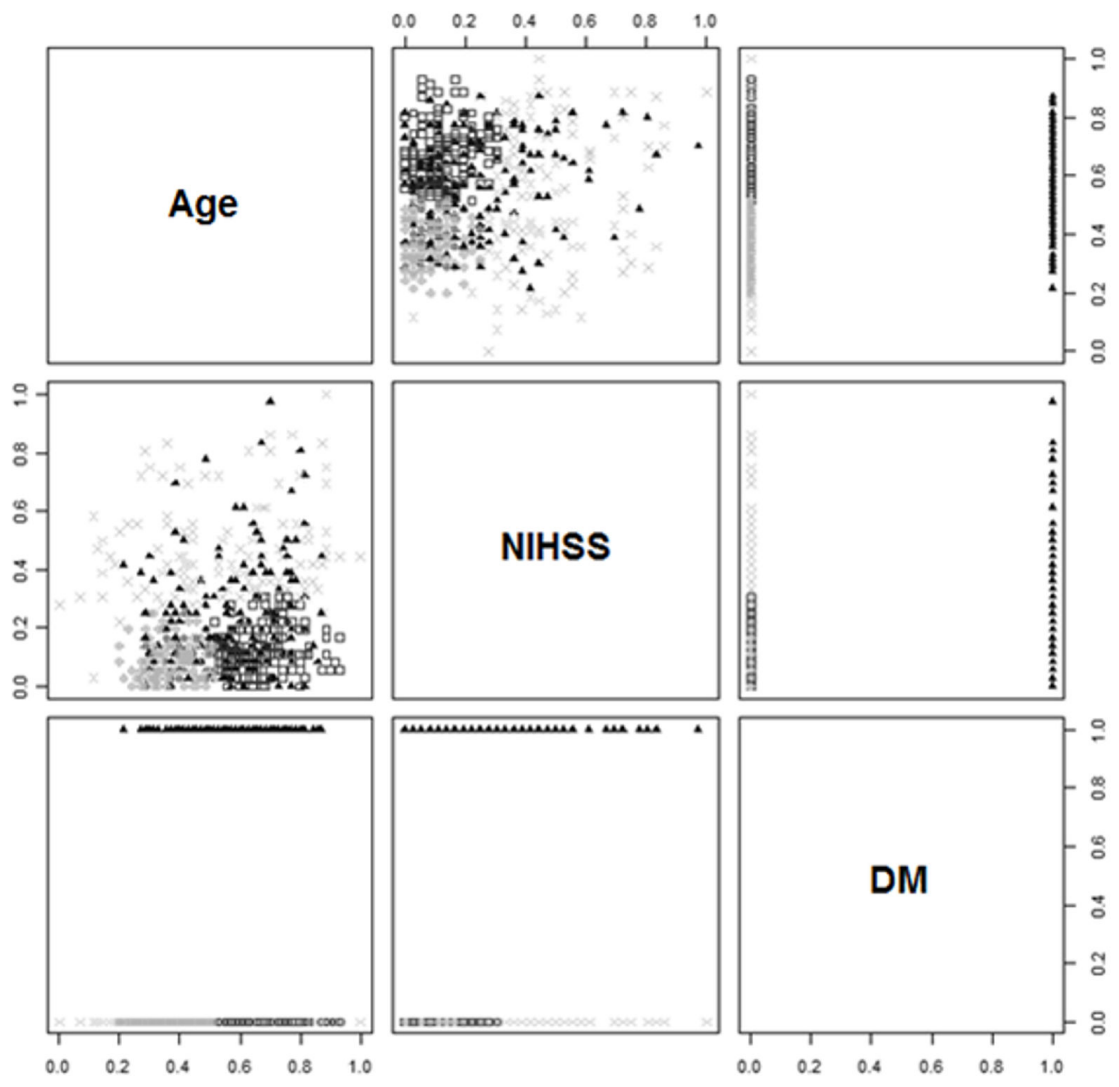
Classification of stroke patients.

The obtained classification of patients (in variable dimensions) based on the expectation-maximization algorithm is shown in Figure 5.

**Figure 6:**
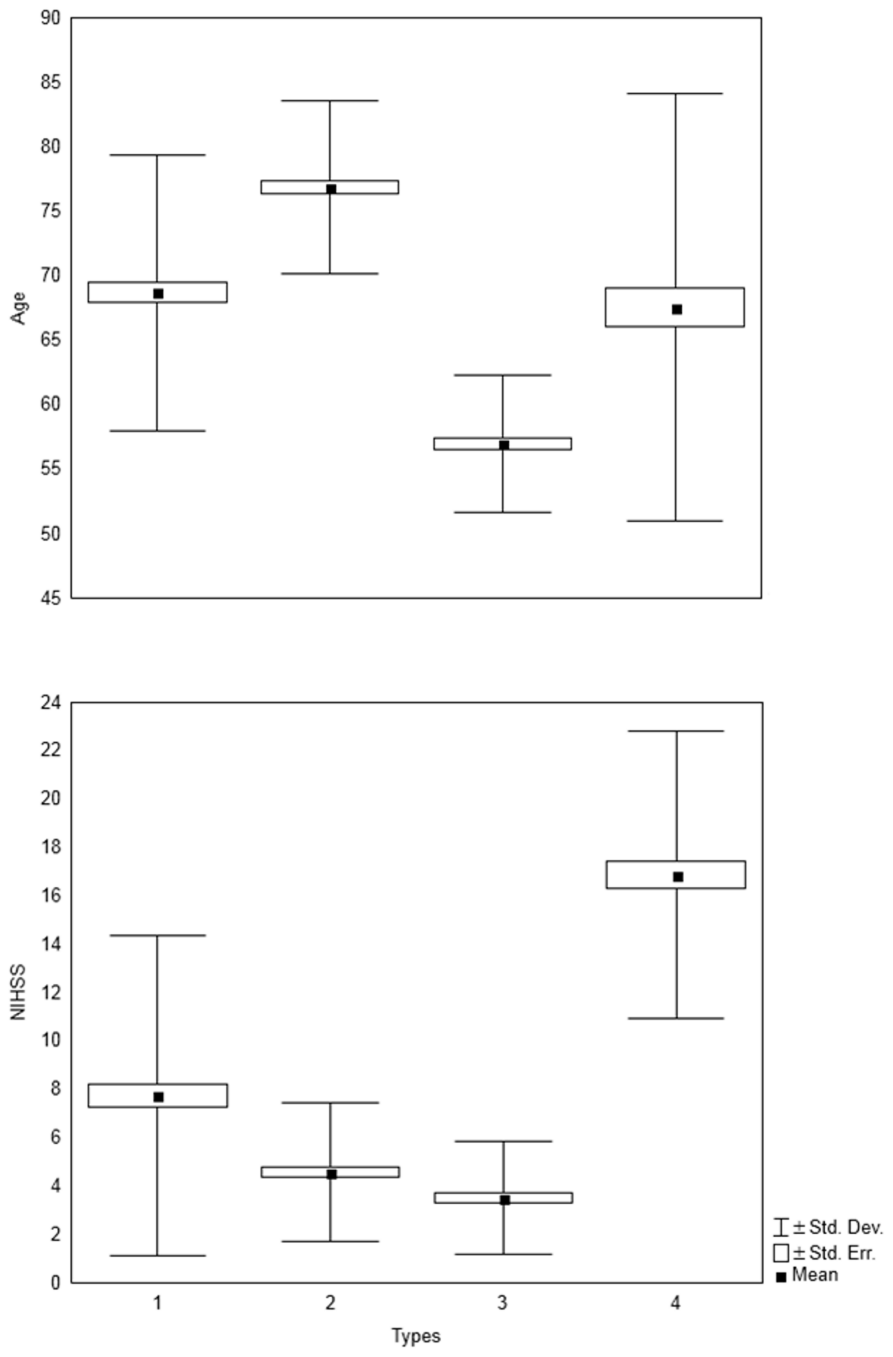
Characteristics of stroke patients following E-M classification (for age and NIHSS).

Comparative statistical characteristics of the types of patients, together with the *F* statistic and *P*s (following one-way ANOVA) using the E-M algorithm, are reported in Table 5 and Figure 6 (the first line in the E-M tables is consistent with the previously given data in the corresponding M-S tables).

**Table 5:** Characteristics of the types of stroke patients (according to the E-M algorithm).

Types	Age mean (SD)	NIHSS mean (SD)	DM status	# of patients
1	68.7 (10.7)	7.8 (6.6)	positive	188
2	76.9 (6.7)	4.6 (2.9)	negative	159
3	57.0 (5.3)	3.5 (2.4)	negative	136
4	67.6 (16.6)	16.9 (6.0)	negative	119
*F* statistic	89.7	192.6	n/a	
*P*	< 0.0001	< 0.0001	n/a	

Based on the F-statistics, a better fit of age in distinguished types was accomplished in the M-S taxonomy, whereas a poorer fit of NIHSS scores was achieved compared to E-M classification. A rough analysis of the characteristics of patients is given in Figure 6 and in Table 6.

**Table 6:** Rough analysis of stroke patients (following E-M classification).

Types	Age	NIHSS	DM status
1	medium	medium	positive
2	older	lower	negative
3	younger	lower	negative
4	medium	higher	negative

**Figure 7:**
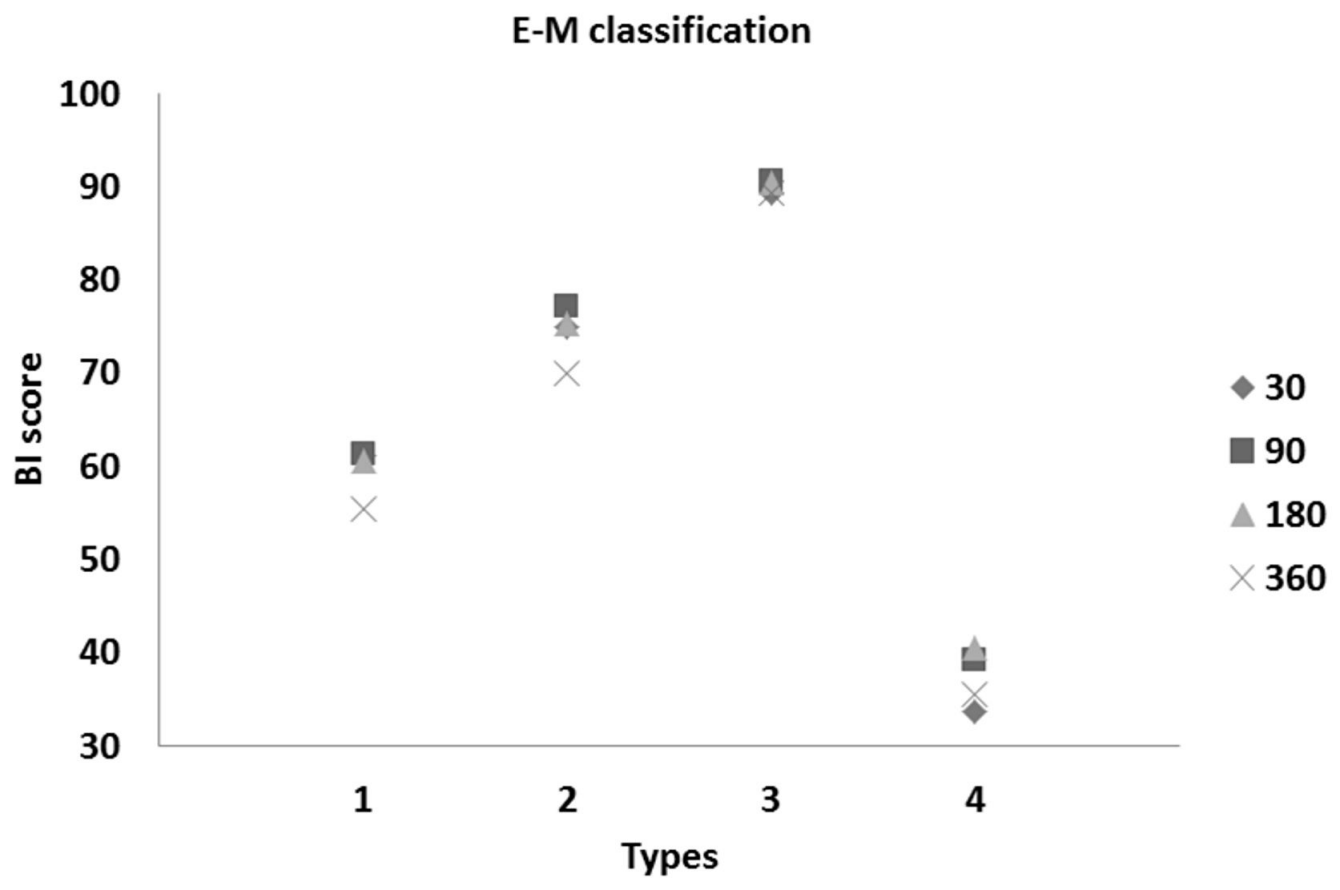
Means of BI scores vs. types of stroke patients at 30, 90, 180, and 360 days after onset of stroke (following the E-M classification).

**Figure 8:**
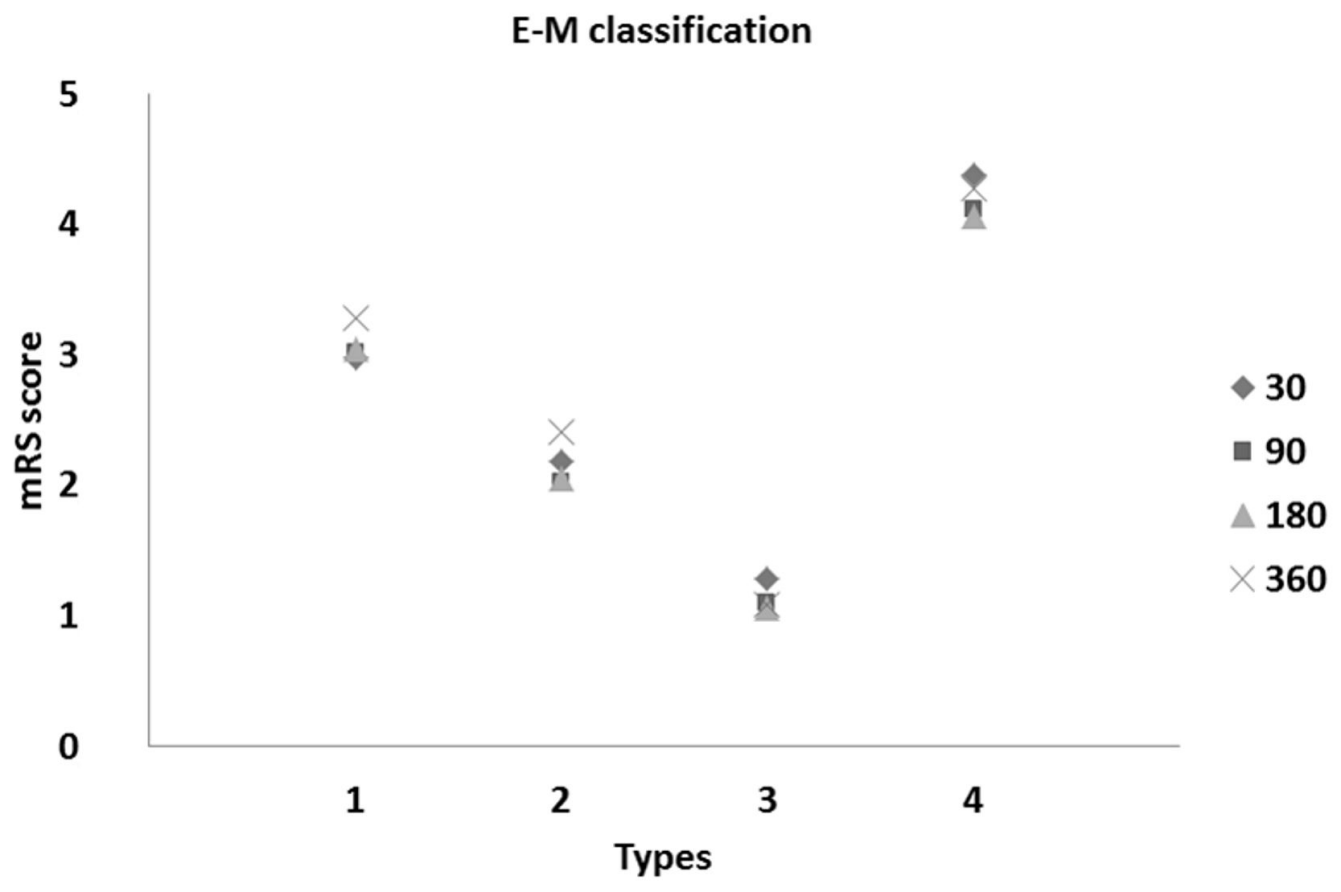
Means of mRS scores vs. types of stroke patients at 30, 90, 180, and 360 days after onset of stroke (following the E-M classification).

Table 6 shows only two differences in assessed levels in comparison to the four types of Table 2. The dissimilarities relate to Type 4 in age and Type 2 in NIHSS, while the choice of DM+ patients was the same for both algorithms. Following the *F* statistics, it can be established that M-S algorithm generated the more statistically significant difference in the means of age, while E-M algorithm in disability. This fact must stand behind different sizes of the clusters for the Types 2, 3, and 4, together with the (minor) dissimilarities in the indicated characteristics of patients. Nevertheless, direct agreement between M-S and E-M classifications was 83%. Moreover, the statistical estimation of the Cohen’s coefficient of agreement [23], κ = 0.766(*P* < 0.0001), provides evidence of highly correlated ratings between the algorithms (i.e., between a simple one and a complex one). The follow-up outcomes based on the E-M classification of patients are reported in Table 7 and Figures 7 and 8.

**Table 7:** Means of follow-up outcomes (based on E-M classification).

Scale	Barthel mean (SD)				mRankin mean (SD)			
Types/Days	30	90	180	360	30	90	180	360
1	61.1 (39.8)	61.3 (40.4)	60.6 (41.0)	55.5 (43.6)	3.0 (2.2)	3.0 (2.2)	3.0 (2.3)	3.3 (2.4)
2	75.0 (32.4)	77.3 (32.8)	75.3 (35.5)	70.0 (37.9)	2.2 (2.0)	2.0 (2.1)	2.1 (2.2)	2.4 (2.2)
3	89.4 (19.3)	90.6 (20.1)	90.4 (22.3)	89.4 (25.5)	1.3 (1.7)	1.1 (1.6)	1.1 (1.7)	1.1 (1.7)
4	33.6 (37.1)	39.2 (42.3)	40.5 (43.3)	35.5 (43.0)	4.4 (1.7)	4.1 (2.1)	4.1 (2.3)	4.3 (2.3)
*F* statistic	59.8	46.7	39.5	34.8	54.5	47.9	43.4	39.4
*P*	< 0.0001	< 0.0001	< 0.0001	< 0.0001	< 0.0001	< 0.0001	< 0.0001	< 0.0001

On the basis of the results given in Table 6, we found a significant difference in means between the taxonomical clusters of patients in follow-up periods since stroke onset (for both the BI and mRS scale outcomes). In comparing follow-up mean values of disability obtained by the M-S algorithm and the E-M classification, the results favor Type 2 patients and predict deterioration of the health status of Type 4 patients (see Table 3 for details). Furthermore, due to a larger number of Type 3 patients, the calculated percentage of patients with the best prognosis increased to 136/602 (22.6%) in E-M classification.

## Discussion

In this paper, we have shown the usefulness of simplified arithmetical assumptions in the identification of certain cohorts of ischemic stroke patients. The proposed solution provides a new possibility for clinical data mining and the explorative assessment of different datasets in epidemiological studies. Compared with the most sophisticated statistical methodology, such as expectation-maximization methodology, the Marczewski-Steinhaus method does not seem to be highly regarded. The strong correlation between the resulting products of the adopted algorithms may convincingly support the performance of taxonomic application in clinical and epidemiological studies. We are not aware of any studies evaluating the M-S method in clinical settings. However, the M-S formula was partially applied in the study of predatory bugs in hazelnut orchards [24].

In our study, the M-S formula is easier in practice, however, we should notice that equally to E-M algorithm it identified only the groups with the best outcome, and it was slightly different in terms of stroke outcomes in groups with medium range of scoring. In the cohort of stroke patients M-S taxonomy revealed only four types of subjects instead of possible 18 types calculated using combination formula. When dealing with rough characteristics of patients, there is a question of why these four particular types of patients are recognized and why the remaining 14 hypothetical combinations of characteristics are absent? Such a phenomenon indicates that general approach to combination does not fit in the clinical settings and most of categories appear in the studied population of stroke patients out of identified categories (e.g. older patients with diabetes, who have very high NIHSS score may not form a cluster, because of high diversity of other factors like heart failure, renal insufficiency, dyselectrolytemia, associated malignancy, inflammation etc.). In addition to hypothetical reflections made above, some interesting conclusions regarding neurological facts emerged in this study. First, in terms of the identification of fairly frequent types of stroke patients with an exceedingly elevated risk of unfavorable outcome at ED admission that is, Type 4 in our study. This cohort consists of middle-aged (46–69 years) stroke patients, which as it was shown in few studies, differs in prognosis. In middle-aged subjects higher percentage for death, recurrent stroke, transient ischemic attack and for coronary event is observed comparing to younger (<45 years) patients [25]. Also poor outcome was more frequent among middle-aged stroke patients and differences in stroke etiology have been identified, when comparing with young subjects [25] from Swedish population. Therefore, Marczewski-Steinhaus approach becomes a novel tool for categorization of patients.

Following the obtained taxonomical outcomes, the strong correlation between the health status at moment of admission to ED and the subsequent recovery of patients is persuasive. However, to predict the outcome of ischemic stroke, an “appropriate” set of risk factors should be taken into account (in our case, age, NIHSS, and DM status were used; among a wide range of risk factors these are have been established as the most important risk factors for stroke and its recovery [13–16]). However, in the preselection phase of the study, a wide range of other risk factors (clinical, demographic, behavioral, environmental, etc.) in different combinations were taken into account. Expectedly, not all factors sets demonstrated similar efficacy in terms of stroke outcomes. Either, no other powerful (comparable) results from a medical point of view were found based on our group of patients. It is of note, that in case of not or poorly “correlated” risk factors, diminutive subgroups of patients arise in the dendrogram. Then, due to a large number of types, the overall characteristics of patients as well as their “correlation” with underling risk factors are not possible to establish. And reversely, the stronger influence of plausible risk factors, the better segregation of patients and their homogeneity in subgroups. In our opinion, to select “appropriate” risk factors, the “classical” statistics could prompt the “novel” approach, and vice versa.

In our study, satisfactorily from statistical and cognitive points of view, the age, NIHSS, and DM status may together affect stroke outcome, and they seem to be the most reliable factors for prognostic purposes. However, a statistical analysis need not stop at the stage of assessment of plausible risk backgrounds and it should continue with further exploration of established datasets. Finally, this statistical approach allows us to extract a subpopulation from the entire group of patients that is characterized by one or more predefined factors and shows similar outcomes. Such a subpopulation is strongly homogenous inside the group. In contrast, the subpopulation differs considerably from the rest of the investigated subpopulations and demonstrates different distances to other subpopulations depending on their characteristics. Such an approach could be useful in different clinical and epidemiological settings.

Even if some findings obtained with the use of this fairly simple statistical device seem to be obvious, the authors hope that it may inspire other investigators to further consider its application in clinical research.

On the basis of the cohort of ischemic stroke patients and adopted statistical methodology, the following conclusions can be reached:

- The Marczewski-Steinhaus metric may provide similar performance to advanced classification methods (such as the expectation-maximization algorithm), which require sophisticated methodological and technical knowledge.
- Promising findings were obtained for stroke patients using this alternative approach, and therefore, novel possibilities are identified in terms of verification of its explorative abilities in other areas.
- Taxonomical ideas could be useful in clinical and epidemiological studies.

## Supporting Information

File S1 (XLSX)
